# Learning together for better health using an evidence-based Learning Health System framework: a case study in stroke

**DOI:** 10.1186/s12916-024-03416-w

**Published:** 2024-05-15

**Authors:** Helena Teede, Dominique A. Cadilhac, Tara Purvis, Monique F. Kilkenny, Bruce C.V. Campbell, Coralie English, Alison Johnson, Emily Callander, Rohan S. Grimley, Christopher Levi, Sandy Middleton, Kelvin Hill, Joanne Enticott

**Affiliations:** 1 Monash Centre for Health Research and Implementation, 43-51 Kanooka Grove, Clayton, VIC Australia; 2 Monash Partners Academic Health Science Centre, 43-51 Kanooka Grove, Clayton, VIC Australia; 3 Stroke and Ageing Research, Department of Medicine, School of Clinical Sciences at Monash Health, Monash University, Level 2 Monash University Research, Victorian Heart Hospital, 631 Blackburn Rd, Clayton, VIC Australia; 4 Stroke Theme, The Florey Institute of Neuroscience and Mental Health, University of Melbourne, Heidelberg, VIC Australia; 5 Department of Neurology, Melbourne Brain Centre, Royal Melbourne Hospital, Parkville, VIC Australia; 6 Department of Medicine, Faculty of Medicine, Dentistry and Health Sciences, University of Melbourne, Victoria, Australia; 7 School of Health Sciences, Heart and Stroke Program, University of Newcastle, Hunter Medical Research Institute, University Drive, Callaghan, NSW Australia; 8 School of Medicine and Dentistry, Griffith University, Birtinya, QLD Australia; 9 Clinical Excellence Division, Queensland Health, Brisbane, Australia; 10 John Hunter Hospital, Hunter New England Local Health District and University of Newcastle, Sydney, NSW Australia; 11 School of Nursing, Midwifery and Paramedicine, Australian Catholic University, Sydney, NSW Australia; 12 Nursing Research Institute, St Vincent’s Health Network Sydney and and Australian Catholic University, Sydney, NSW Australia; 13 Stroke Foundation, Level 7, 461 Bourke St, Melbourne, VIC Australia

**Keywords:** Learning Health System, Stroke, Evidence-based medicine, Person-centred care, Models of care, Healthcare improvement

## Abstract

**Background:**

In the context of expanding digital health tools, the health system is ready for Learning Health System (LHS) models. These models, with proper governance and stakeholder engagement, enable the integration of digital infrastructure to provide feedback to all relevant parties including clinicians and consumers on performance against best practice standards, as well as fostering innovation and aligning healthcare with patient needs. The LHS literature primarily includes opinion or consensus-based frameworks and lacks validation or evidence of benefit. Our aim was to outline a rigorously codesigned, evidence-based LHS framework and present a national case study of an LHS-aligned national stroke program that has delivered clinical benefit.

**Main text:**

Current core components of a LHS involve capturing evidence from communities and stakeholders (quadrant 1), integrating evidence from research findings (quadrant 2), leveraging evidence from data and practice (quadrant 3), and generating evidence from implementation (quadrant 4) for iterative system-level improvement. The Australian Stroke program was selected as the case study as it provides an exemplar of how an iterative LHS works in practice at a national level encompassing and integrating evidence from all four LHS quadrants. Using this case study, we demonstrate how to apply evidence-based processes to healthcare improvement and embed real-world research for optimising healthcare improvement. We emphasize the transition from research as an endpoint, to research as an enabler and a solution for impact in healthcare improvement.

**Conclusions:**

The Australian Stroke program has nationally improved stroke care since 2007, showcasing the value of integrated LHS-aligned approaches for tangible impact on outcomes. This LHS case study is a practical example for other health conditions and settings to follow suit.

## Background

Internationally, health systems are facing a crisis, driven by an ageing population, increasing complexity, multi-morbidity, rapidly advancing health technology and rising costs that threaten sustainability and mandate transformation and improvement [1, 2]. Although research has generated solutions to healthcare challenges, and the advent of big data and digital health holds great promise, entrenched siloes and poor integration of knowledge generation, knowledge implementation and healthcare delivery between stakeholders, curtails momentum towards, and consistent attainment of, evidence-and value-based care [3]. This is compounded by the short supply of research and innovation leadership within the healthcare sector, and poorly integrated and often inaccessible health data systems, which have crippled the potential to deliver on digital-driven innovation [4]. Current approaches to healthcare improvement are also often isolated with limited sustainability, scale-up and impact [5].

**Fig. 1:**
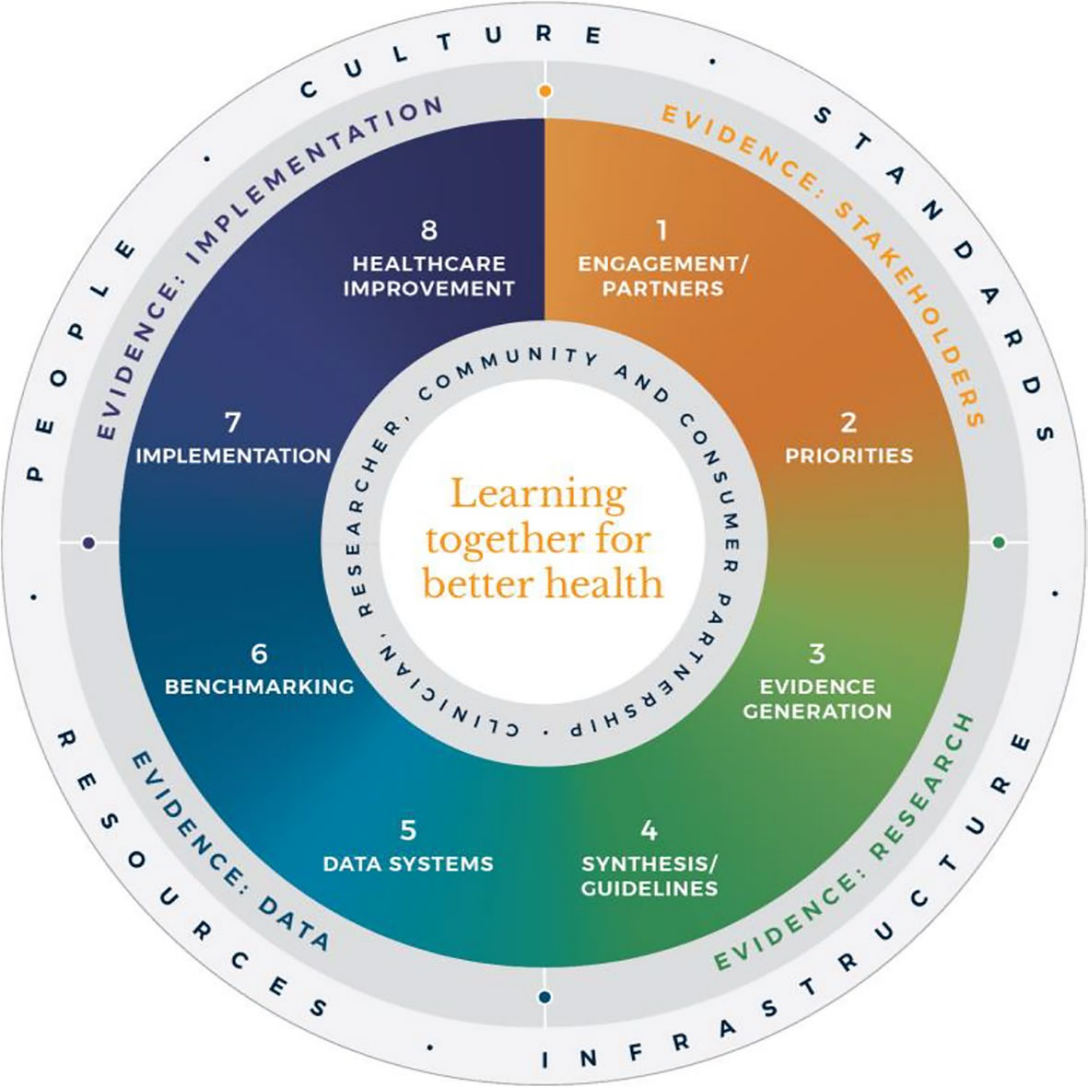
Monash Learning Health System: The Learn Together for Better Health Framework developed by Monash Partners and Monash University (from Enticott et al. 2021 [7]). Four evidence quadrants: Q1 (orange) is evidence from stakeholders; Q2 (green) is evidence from research; Q3 (light blue) is evidence from data; and, Q4 (dark blue) is evidence from implementation and healthcare improvement

Evidence suggests that integration and partnership across academic and healthcare delivery stakeholders are key to progress, including those with lived experience and their families (referred to here as consumers and community), diverse disciplines (both research and clinical), policy makers and funders. Utilization of evidence from research and evidence from practice including data from routine care, supported by implementation research, are key to sustainably embedding improvement and optimising health care and outcomes. A strategy to achieve this integration is through the *Learning Health System (LHS)* (Fig. 1) [2, 6–8]. Although there are numerous publications on LHS approaches [9–12], many focus on research perspectives and data, most do not demonstrate tangible healthcare improvement or better health outcomes. [6]

In developed nations, it has been estimated that 60% of care provided aligns with the evidence base, 30% is low value and 10% is potentially harmful [13]. In some areas, clinical advances have been rapid and research and evidence have paved the way for dramatic improvement in outcomes, mandating rapid implementation of evidence into healthcare (e.g. polio and COVID-19 vaccines). However, healthcare improvement is challenging and slow [5]. Health systems are highly complex in their design, networks and interacting components, and change is difficult to enact, sustain and scale up. [3] New effective strategies are needed to meet community needs and deliver evidence-based and value-based care, which reorients care from serving the provider, services and system, towards serving community needs, based on evidence and quality. It goes beyond cost to encompass patient and provider experience, quality care and outcomes, efficiency and sustainability [2, 6].

The costs of stroke care are expected to rise rapidly in the next decades, unless improvements in stroke care to reduce the disabling effects of strokes can be successfully developed and implemented [14]. Here, we briefly describe the Monash LHS framework (Fig. 1) [2, 6, 7] and outline an exemplar case in order to demonstrate how to apply evidence-based processes to healthcare improvement and embed real-world research for optimising healthcare. The Australian LHS exemplar in stroke care has driven nationwide improvement in stroke care since 2007.

### An evidence-based Learning Health System framework

In Australia, members of this author group (HT, AJ, JE) have rigorously co-developed an evidence-based LHS framework, known simply as the Monash LHS [7]. The Monash LHS was designed to support sustainable, iterative and continuous robust benefit of improved clinical outcomes. It was created with national engagement in order to be applicable to Australian settings. Through this rigorous approach, core LHS principles and components have been established (Fig. 1). Evidence shows that people/workforce, culture, standards, governance and resources were all key to an effective LHS [2, 6]. Culture is vital including trust, transparency, partnership and co-design. Key processes include legally compliant data sharing, linkage and governance, resources, and infrastructure [4]. The Monash LHS integrates disparate and often siloed stakeholders, infrastructure and expertise to ‘Learn Together for Better Health’ [7] (Fig. 1). This integrates (i) evidence from community and stakeholders including priority areas and outcomes; (ii) evidence from research and guidelines; (iii) evidence from practice (from data) with advanced analytics and benchmarking; and (iv) evidence from implementation science and health economics. Importantly, it starts with the problem and priorities of key stakeholders including the community, health professionals and services and creates an iterative learning system to address these. The following case study was chosen as it is an exemplar of how a Monash LHS-aligned national stroke program has delivered clinical benefit.

#### Australian Stroke Learning Health System

Internationally, the application of LHS approaches in stroke has resulted in improved stroke care and outcomes [12]. For example, in Canada a sustained decrease in 30-day in-hospital mortality has been found commensurate with an increase in resources to establish the multifactorial stroke system intervention for stroke treatment and prevention [15]. Arguably, with rapid advances in evidence and in the context of an ageing population with high cost and care burden and substantive impacts on quality of life, stroke is an area with a need for rapid research translation into evidence-based and value-based healthcare improvement. However, a recent systematic review found that the existing literature had few comprehensive examples of LHS adoption [12]. Although healthcare improvement systems and approaches were described, less is known about patient-clinician and stakeholder engagement, governance and culture, or embedding of data informatics into everyday practice to inform and drive improvement [12]. For example, in a recent review of quality improvement collaborations, it was found that although clinical processes in stroke care are improved, their short-term nature means there is uncertainty about sustainability and impacts on patient outcomes [16]. Table 1 provides the main features of the Australian Stroke LHS based on the four core domains and eight elements of the Learning Together for Better Health Framework described in Fig. 1. The features are further expanded on in the following sections.

**Table 1:** Core features of the Australian Stroke Learning Health System (LHS). Each row provides an example of what was undertaken to inform the design or establishment of different features of the Australian Stroke LHS. It is not an exhaustive description and some features may have had a role in other components

Learning Health System framework^a^	Stakeholders	Research	Data	Implementation
	**Evidence quadrant 1**	**Evidence quadrant 2**	**Evidence quadrant 3**	**Evidence quadrant 4**
	**1) Engagement partners** **2) Identifying priorities**	**3) Evidence-based information** **4) Evidence synthesis**	**5) Data and information Systems** **6) Benchmarking**	**7) Implementation** **8) Healthcare improvement**
**Establishing national standards of acute stroke care and data systems to enable standardised monitoring and feedback to clinicians**	Co-designed with clinical experts, academics and people with lived experience by the Australian Commission on Safety and Quality in Health Care	Australian Acute Stroke Clinical Care Standards developed in 2015 [17] and reviewed in 2019 [18]	Australian Stroke Data Tool developed to share data for the same patients for overlapping variables from the Australian Stroke Clinical Registry (AuSCR) and the National Stroke Audit program [19]	Reporting of Acute Stroke Care Standard in the AuSCR annual reports (partial set), and biennial Audit program reports (full set). Hospital provided with individual performance against national benchmarks
**Clinical practice guidelines and data systems to enable standardised monitoring and feedback to clinicians**	Interdisciplinary clinical and academic leadership Partnership with systematic review experts (i.e. Cochrane) Strong support and leadership from national peak advocacy body (Stroke Foundation)	Stroke Clinical Guidelines updated in 2017 and transitioned from 2018 to the first example globally of a ‘living guidelines’ approach available as an online reference standard for stroke care [20, 21].	Comprehensive audit program and clinical quality registry that provide tailored reports back to participating hospitals as well as national summary reports of performance for advocacy	Stroke Foundation InformMe website (https://informme.org.au/) designed for clinicians to access their personalised audit reports and other dedicated resources for health professionals to improve stroke care
**Support for site-level quality improvement activities**	Site champions, clinical leaders and coordinators Communities of Practice with embedded academics, and clinical networks integrating with health departments	Local evidence of practice gaps	Benchmarked reports of performance, quality improvement projects e.g. based on plan-do-study-act cycles [22, 23]	Pre-post data reviews
**Implementation research to improve the evidence for effective quality improvement strategies**	Academic interdisciplinary collaborations facilitated through grants	Implementation and improvement science research	Stand-alone dataset or based on national data that are routinely collected + / − augmentation with data linkage	Adoption in policy or practice, clinical guidelines evidence synthesis

^a^Based on the Monash Learning Health System developed by Monash Partners and Monash University, shown in Fig. 1

### *Evidence from stakeholders (LHS quadrant 1, *Fig. 1*)*

#### Engagement, partners and priorities

Within the stroke field, there have been various support mechanisms to facilitate an LHS approach including partnership and broad stakeholder engagement that includes clinical networks and policy makers from different jurisdictions. Since 2008, the Australian Stroke Coalition has been co-led by the Stroke Foundation, a charitable consumer advocacy organisation, and Stroke Society of Australasia a professional society with membership covering academics and multidisciplinary clinician networks, that are collectively working to improve stroke care (https://australianstrokecoalition.org.au/). Surveys, focus groups and workshops have been used for identifying priorities from stakeholders. Recent agreed priorities have been to improve stroke care and strengthen the voice for stroke care at a national (https://strokefoundation.org.au/) and international level (https://www.world-stroke.org/news-and-blog/news/world-stroke-organization-tackle-gaps-in-access-to-quality-stroke-care), as well as reduce duplication amongst stakeholders. This activity is built on a foundation and culture of research and innovation embedded within the stroke ‘community of practice’. Consumers, as people with lived experience of stroke are important members of the Australian Stroke Coalition, as well as representatives from different clinical colleges. Consumers also provide critical input to a range of LHS activities via the Stroke Foundation Consumer Council, Stroke Living Guidelines committees, and the Australian Stroke Clinical Registry (AuSCR) Steering Committee (described below).

### *Evidence from research (LHS quadrant 2, *Fig. 1*)*

#### Advancement of the evidence for stroke interventions and synthesis into clinical guidelines

To implement best practice, it is crucial to distil the large volume of scientific and trial literature into actionable recommendations for clinicians to use in practice [24]. The first Australian clinical guidelines for acute stroke were produced in 2003 following the increasing evidence emerging for prevention interventions (e.g. carotid endarterectomy, blood pressure lowering), acute medical treatments (intravenous thrombolysis, aspirin within 48 h of ischemic stroke), and optimised hospital management (care in dedicated stroke units by a specialised and coordinated multidisciplinary team) [25]. Importantly, a number of the innovations were developed, researched and proven effective by key opinion leaders embedded in the Australian stroke care community. In 2005, the clinical guidelines for Stroke Rehabilitation and Recovery [26] were produced, with subsequent merged guidelines periodically updated. However, the traditional process of periodic guideline updates is challenging for end users when new research can render recommendations redundant and this lack of currency erodes stakeholder trust [27]. In response to this challenge the Stroke Foundation and Cochrane Australia entered a pioneering project to produce the first electronic ‘living’ guidelines globally [20]. Major shifts in the evidence for reperfusion therapies (e.g. extended time-window intravenous thrombolysis and endovascular clot retrieval), among other advances, were able to be converted into new recommendations, approved by the Australian National Health and Medical Research Council within a few months of publication. Feedback on this process confirmed the increased use and trust in the guidelines by clinicians. The process informed other living guidelines programs, including the successful COVID-19 clinical guidelines [28].

However, best practice clinical guideline recommendations are necessary but insufficient for healthcare improvement and nesting these within an LHS with stakeholder partnership, enables implementation via a range of proven methods, including audit and feedback strategies [29].

### *Evidence from data and practice (LHS quadrant 3, *Fig. 1*)*

**Fig. 2:**
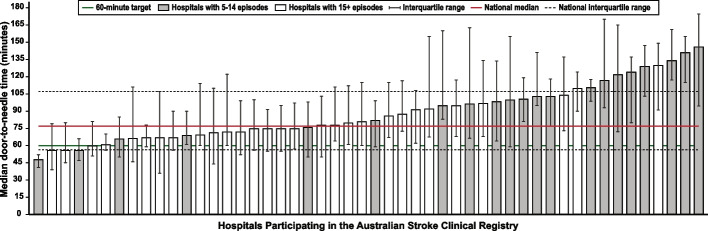
Example performance report from the Australian Stroke Clinical Registry: average door-to-needle time in providing intravenous thrombolysis by different hospitals in 2021 [36]. Each bar in the figure represents a single hospital

*Data systems and benchmarking*: revealing the disparities in care between health services. A national system for standardized stroke data collection was established as the National Stroke Audit program in 2007 by the Stroke Foundation [30] following various state-level programs (e.g. New South Wales Audit) [31] to identify evidence-practice gaps and prioritise improvement efforts to increase access to stroke units and other acute treatments [32]. The Audit program alternates each year between acute (commencing in 2007) and rehabilitation in-patient services (commencing in 2008). The Audit program provides a ‘deep dive’ on the majority of recommendations in the clinical guidelines whereby participating hospitals provide audits of up to 40 consecutive patient medical records and respond to a survey about organizational resources to manage stroke. In 2009, the AuSCR was established to provide information on patients managed in acute hospitals based on a small subset of quality processes of care linked to benchmarked reports of performance (Fig. 2) [33]. In this way, the continuous collection of high-priority processes of stroke care could be regularly collected and reviewed to guide improvement to care [34]. Plus clinical quality registry programs within Australia have shown a meaningful return on investment attributed to enhanced survival, improvements in quality of life and avoided costs of treatment or hospital stay [35].

**Fig. 3:**
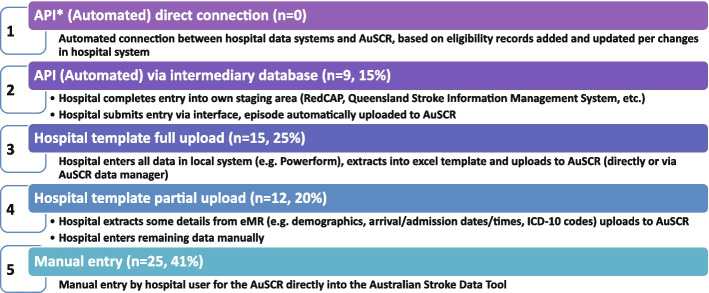
Current status of automated data importing solutions in the Australian Stroke Clinical Registry, 2022, with ‘*n*’ representing the number of hospitals. AuSCR, Australian Stroke Clinical Registry; AuSDaT, Australian Stroke Data Tool; API, Application Programming Interface; ICD, International Classification of Diseases; RedCAP, Research Electronic Data Capture; eMR, electronic medical records

The Australian Stroke Coalition endorsed the creation of an integrated technological solution for collecting data through a single portal for multiple programs in 2013. In 2015, the Stroke Foundation, AuSCR consortium, and other relevant groups cooperated to design an integrated data management platform (the Australian Stroke Data Tool) to reduce duplication of effort for hospital staff in the collection of overlapping variables in the same patients [19]. Importantly, a national data dictionary then provided the common data definitions to facilitate standardized data capture. Another important feature of AuSCR is the collection of patient-reported outcome surveys between 90 and 180 days after stroke, and annual linkage with national death records to ascertain survival status [33]. To support a LHS approach, hospitals that participate in AuSCR have access to a range of real-time performance reports. In efforts to minimize the burden of data collection in the AuSCR, interoperability approaches to import data directly from hospital or state-level managed stroke databases have been established (Fig. 3); however, the application has been variable and 41% of hospitals still manually enter all their data.

For acute stroke care, the Australian Commission on Quality and Safety in Health Care facilitated the co-design (clinicians, academics, consumers) and publication of the national Acute Stroke Clinical Care Standard in 2015 [17], and subsequent review [18]. The indicator set for the Acute Stroke Standard then informed the expansion of the minimum dataset for AuSCR so that hospitals could routinely track their performance. The national Audit program enabled hospitals not involved in the AuSCR to assess their performance every two years against the Acute Stroke Standard. Complementing these efforts, the Stroke Foundation, working with the sector, developed the Acute and Rehabilitation Stroke Services Frameworks to outline the principles, essential elements, models of care and staffing recommendations for stroke services (https://informme.org.au/guidelines/national-stroke-services-frameworks). The Frameworks are intended to guide where stroke services should be developed, and monitor their uptake with the organizational survey component of the Audit program.

### *Evidence from implementation and healthcare improvement (LHS quadrant 4, *Fig. 1*)*

Research to better utilize and augment data from registries through linkage [37–40] and to ensure presentation of hospital or service level data are understood by clinicians has ensured advancement in the field for the Australian Stroke LHS [41]. Importantly, greater insights into whole patient journeys, before and after a stroke, can now enable exploration of value-based care. The LHS and stroke data platform have enabled focused and time-limited projects to create a better understanding of the quality of care in acute or rehabilitation settings [22, 42, 43]. Within stroke, all the elements of an LHS culminate into the ready availability of benchmarked performance data and support for implementation of strategies to address gaps in care.

Implementation research to grow the evidence base for effective improvement interventions has also been a key pillar in the Australian context. These include multi-component implementation interventions to achieve behaviour change for particular aspects of stroke care, [22, 23, 44, 45] and real-world approaches to augmenting access to hyperacute interventions in stroke through the use of technology and telehealth [46–49]. The evidence from these studies feeds into the living guidelines program and the data collection systems, such as the Audit program or AuSCR, which are then amended to ensure data aligns to recommended care. For example, the use of ‘hyperacute aspirin within the first 48 h of ischemic stroke’ was modified to be ‘hyperacute antiplatelet…’ to incorporate new evidence that other medications or combinations are appropriate to use. Additionally, new datasets have been developed to align with evidence such as the Fever, Sugar, and Swallow variables [42]. Evidence on improvements in access to best practice care from the acute Audit program [50] and AuSCR is emerging [36]. For example, between 2007 and 2017, the odds of receiving intravenous thrombolysis after ischemic stroke increased by 16% 9OR 1.06 95% CI 1.13–1.18) and being managed in a stroke unit by 18% (OR 1.18 95% CI 1.17–1.20). Over this period, the median length of hospital stay for all patients decreased from 6.3 days in 2007 to 5.0 days in 2017 [51]. When considering the number of additional patients who would receive treatment in 2017 in comparison to 2007 it was estimated that without this additional treatment, over 17,000 healthy years of life would be lost in 2017 (17,786 disability-adjusted life years) [51]. There is evidence on the cost-effectiveness of different system-focussed strategies to augment treatment access for acute ischemic stroke (e.g. Victorian Stroke Telemedicine program [52] and Melbourne Mobile Stroke Unit ambulance [53]). Reciprocally, evidence from the national Rehabilitation Audit, where the LHS approach has been less complete or embedded, has shown fewer areas of healthcare improvement over time [51, 54].

Within the field of stroke in Australia, there is indirect evidence that the collective efforts that align to establishing the components of a LHS have had an impact. Overall, the age-standardised rate of stroke events has reduced by 27% between 2001 and 2020, from 169 to 124 events per 100,000 population. Substantial declines in mortality rates have been reported since 1980. Commensurate with national clinical guidelines being updated in 2007 and the first National Stroke Audit being undertaken in 2007, the mortality rates for men (37.4 deaths per 100,000) and women (36.1 deaths per 100,0000 has declined to 23.8 and 23.9 per 100,000, respectively in 2021 [55].

Underpinning the LHS with the integration of the four quadrants of evidence from stakeholders, research and guidelines, practice and implementation, and core LHS principles have been addressed. Leadership and governance have been important, and programs have been established to augment workforce training and capacity building in best practice professional development. Medical practitioners are able to undertake courses and mentoring through the Australasian Stroke Academy (http://www.strokeacademy.com.au/) while nurses (and other health professionals) can access teaching modules in stroke care from the Acute Stroke Nurses Education Network (https://asnen.org/). The Association of Neurovascular Clinicians offers distance-accessible education and certification to develop stroke expertise for interdisciplinary professionals, including advanced stroke co-ordinator certification (www.anvc.org). Consumer initiative interventions are also used in the design of the AuSCR Public Summary Annual reports (available at https://auscr.com.au/about/annual-reports/) and consumer-related resources related to the Living Guidelines (https://enableme.org.au/resources).

The important success factors and lessons from stroke as a national exemplar LHS in Australia include leadership, culture, workforce and resources integrated with (1) established and broad partnerships across the academic-clinical sector divide and stakeholder engagement; (2) the living guidelines program; (3) national data infrastructure, including a national data dictionary that provides the common data framework to support standardized data capture; (4) various implementation strategies including benchmarking and feedback as well as engagement strategies targeting different levels of the health system; and (5) implementation and improvement research to advance stroke systems of care and reduce unwarranted variation in practice (Fig. 1). Priority opportunities now include the advancement of interoperability with electronic medical records as an area all clinical quality registry’s programs needs to be addressed, as well as providing more dynamic and interactive data dashboards tailored to the need of clinicians and health service executives.

## Conclusions

There is a clear mandate to optimise healthcare improvement with big data offering major opportunities for change. However, we have lacked the approaches to capture evidence from the community and stakeholders, to integrate evidence from research, to capture and leverage data or evidence from practice and to generate and build on evidence from implementation using iterative system-level improvement. The LHS provides this opportunity and is shown to deliver impact. Here, we have outlined the process applied to generate an evidence-based LHS and provide a leading exemplar in stroke care. This highlights the value of moving from single-focus isolated approaches/initiatives to healthcare improvement and the benefit of integration to deliver demonstrable outcomes for our funders and key stakeholders — our community. This work provides insight into strategies that can both apply evidence-based processes to healthcare improvement as well as implementing evidence-based practices into care, moving beyond research as an endpoint, to research as an enabler, underpinning delivery of better healthcare.

## Data Availability

Not applicable

## Abbreviations

AuSCR: Australian Stroke Clinical Registry
CI: Confidence interval
LHS: Learning Health System
OR: Odds ratio
